# Fractal Geometry Illustrated Left Atrial Appendage Morphology That Predicted Thrombosis and Stroke in Patients With Atrial Fibrillation

**DOI:** 10.3389/fcvm.2022.779528

**Published:** 2022-05-10

**Authors:** Chuxiang Lei, Qi Gao, Runjie Wei, Qijie Li, Xingli Liu, Lingmin Wu, Yan Yao, Hongguang Fan, Zhe Zheng

**Affiliations:** ^1^State Key Laboratory of Cardiovascular Diseases, Department of Cardiac Surgery, National Center for Cardiovascular Diseases, Fuwai Hospital, Peking Union Medical College and Chinese Academy of Medical Sciences, Beijing, China; ^2^School of Aeronautics and Astronautics, Institute of Fluid Engineering, Zhejiang University, Hangzhou, China; ^3^Hangzhou Shengshi Technology Co., Ltd., Hangzhou, China

**Keywords:** left atrial appendage, fractal dimension, morphological complexity, thrombosis, atrial fibrillation

## Abstract

**Background:**

This study aims to correlate the morphological complexity of left atrial appendage (LAA) with thrombosis and stroke in patients with atrial fibrillation (AF).

**Methods:**

The training cohort consisted of 46 patients with AF (age 55.8 ± 7.2 years, 73.9% men) who were referred for radiofrequency catheter ablation. An independent validation cohort consisting of 443 patients with AF was enrolled for further verification. All patients in the training cohort underwent both transesophageal echocardiography (TEE) and enhanced computed tomography (CT). Fractal dimension (FD) analysis was performed to evaluate the morphological complexity of LAAs quantitatively. Clinical and imaging manifestations, FD of LAAs, and diagnostic accuracy were investigated and compared between patients with AF in both training and validation cohorts.

**Results:**

In the training cohort, LAAs (*n* = 22) with thrombi had significantly higher FD than those without thrombi (*n* = 24) h 0.44 ± 0.07 vs. 2.35 ± 0.11, *p* = 0.003). Receiver-operating characteristic (ROC) analysis suggested that the diagnostic accuracy of FD combined with a CHA_2_DS_2_-VaSc score was significantly higher than that of the CHA_2_DS_2_-VaSc score alone in low- to moderate-risk patients with AF (area under the curve 0.8479 vs. 0.6958, *p* = 0.009). The results were also validated in an independent external validation cohort and demonstrated that increased FD was associated with stroke. Hemodynamic analysis revealed that LAAs with thrombi and high FD were prone to blood stasis and lower blood flow rate.

**Conclusion:**

LAA morphological complexity is closely associated with thrombosis and stroke in patients with paroxysmal AF. A new risk assessment system combining CHA_2_DS_2_-VaSc score and FD has a higher diagnostic accuracy in predicting LAA thrombosis.

## Introduction

Atrial fibrillation (AF) is the most common clinically significant cardiac arrhythmia characterized by the presence of irregular fibrillatory waves. The incidence of AF increases markedly with age and approximately doubles with each decade (1, 2). AF is associated with an increased risk of thrombosis in the left atrial (LA), and it has been reported that the left atrial appendage (LAA) is suspected to be a vital source of cerebral emboli and may lead to ischemic stroke (3, 4). Previous studies suggested that, among all cardiogenic strokes, more than 90% of the emboli were from LAA. Furthermore, the risk of stroke increases nearly fivefold when AF is present, and so does the mortality in patients with AF and thrombosis (5).

According to Virchow’s triad, thrombosis is determined by stasis of blood flow, endothelial injury, and hypercoagulability. At present, the CHA_2_DS_2_-VaSc score is the most widely used model for assessing the risk of cerebrovascular events. This index includes age, congestive heart failure, sex, diabetes, hypertension, stroke, and vascular disease. Although it covers the assessment of the endothelial function and coagulability status, it does not reflect the blood flow stasis in LA or LAA. When AF occurs, LAA fails to contract effectively and leads to slow blood flow or stasis in LAA, which eventually results in thrombosis. It is obvious that the morphology of LAA has a significant impact on the blood flow. In contrast, studies suggest that the shape and structure of LAA are complex and diverse among the population. Cardiac computed tomography (CT) imaging has been widely used in the evaluation of cardiovascular diseases, including heart structures evaluation, calcium score in risk stratification, and coronary artery disease assessment (6, 7). For example, the coronary artery calcium score, which is readily detected on CT images, represents a surrogate marker of the presence and extent of coronary artery disease (8). In addition, cardiac CT is used to analyze the morphology and function of the heart. Several studies reported that the risk score based on cardiac CT could categorize disease severity and predict outcomes, including mitral annular and coronary artery calcification (7, 9). Moreover, multidetector CT scan image LAA at high resolution (10), and the existing classification criteria of LAA morphology according to these images are used to describe the anatomical shapes (5). However, none of them are able to characterize the internal morphological features. Therefore, the blood flow stasis is extremely difficult to assess.

To quantitatively describe the morphological complexity of LAA, we introduced the box-count dimension in the fractal geometry. Fractal dimension (FD) is the most crucial concept and content in the fractal geometry theory. Box-counting dimension is a commonly used parameter in FD, where it can evaluate the complexity and irregularity of a real object or a fractal object quantitatively. Fractal geometry analysis has been applied in many fields such as the dimensionless measurement of trabeculation complexity. Furthermore, it has been used to evaluate the left ventricular (LV) trabeculation quantitatively in LV non-compaction patients and define the normal FD range of LV trabeculation (11). Similarly, the fractal analysis can assess the correlation between the complexity of right ventricle trabecula and hemodynamic parameters in patients with pulmonary hypertension and identify retinal microvascular changes in the early stage of patients with type 2 diabetes (11–13). Our study first applied FD calculated by enhanced CT image of LAA to evaluate its complexity and further revealed the association between thrombosis and shape complexity of LAA.

## Materials and Methods

### Patients’ Enrollment

**FIGURE 1:**
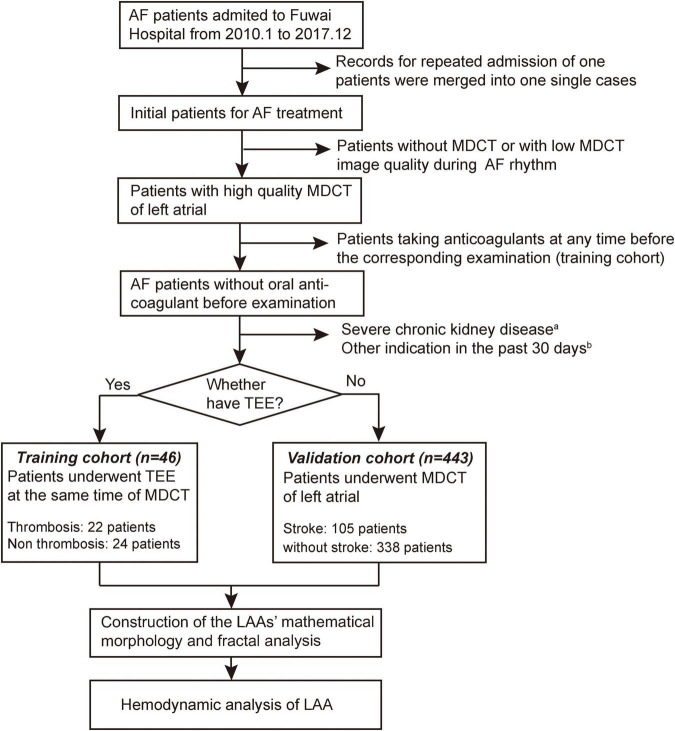
Flowchart of the study. (a) Stage 4 or 5 or end-stage chronic kidney disease. (b) Deep vein thrombosis/pulmonary embolism, hip/knee replacement, femur/tibia/patella fracture, thrombectomy, or chronic hypercoagulable state.

This is a retrospective cohort study that included all consecutive patients with AF who were first admitted to Fuwai Hospital and scheduled to undergo radiofrequency ablation from January 2010 to December 2017. According to the 2020 European Society of Cardiology guideline (14), the diagnosis of AF was determined by a standard 12-lead ECG showing heart rhythm with no discernible repeating P waves and irregular RR intervals. All the patients enrolled in this study should have undergone the multidetector computed tomography (MDCT) of the LA. For patients with paroxysmal AF, the CT images used for 3D reconstruction were collected during the diastolic phase, whereas for patients with persistent AF, the images were collected during the whole cardiac cycle due to the inability to distinguish between systolic and diastolic phases. Transesophageal echocardiography (TEE) was also required for patients in the training cohort to determine the presence of LAA thrombosis. In the training cohort, all patients did not take anticoagulants at any time before the corresponding examination and had their imaging examination performed before the anticoagulation treatment they would have undergone prior to radiofrequency ablation. An independent external validation cohort consisted of patients with AF who were examined by LA MDCT over the same period of time. Similar inclusion criteria, except for the use of anticoagulants and TEE examination, were applied for these patients (not receiving regular anticoagulation within 1 year prior to imaging), and these patients were divided into two groups based on whether they had the stroke. Patients with low MDCT image quality during AF rhythm were excluded, as well as patients who had undergone MDCT without contrast enhancement because of renal dysfunction. Other exclusive criteria included organized thrombus in LAA, severe chronic kidney disease (stage 4 or 5 or end-stage chronic kidney disease), other anticoagulant indications in the past 30 days (including deep vein thrombosis/pulmonary embolism, hip/knee replacement, femur/tibia/patella fracture, thrombectomy, or chronic hypercoagulable state), and poor CT or echocardiographic image quality. Eventually, a total of 46 patients were enrolled in the training cohort, and 443 patients were assigned to validation cohort. The overall flowchart of this study is shown in Figure 1. Approval to conduct this study was obtained from the Institutional Review Board, Fuwai Hospital. All patients signed an informed consent form using their clinical data for research, and patients were not involved in the study design, data analysis, and other research processes. All the data used in this study were necessary examinations for patients.

### Echocardiographic Data Analysis

See Supplementary Material.

### Cardiac Computed Tomography Scan and Image Analysis

See Supplementary Material.

### Classification of Left Atrial Appendages’ Morphology

See Supplementary Material.

### Construction of Left Atrial Appendages’ Mathematical Morphology

**FIGURE 2:**
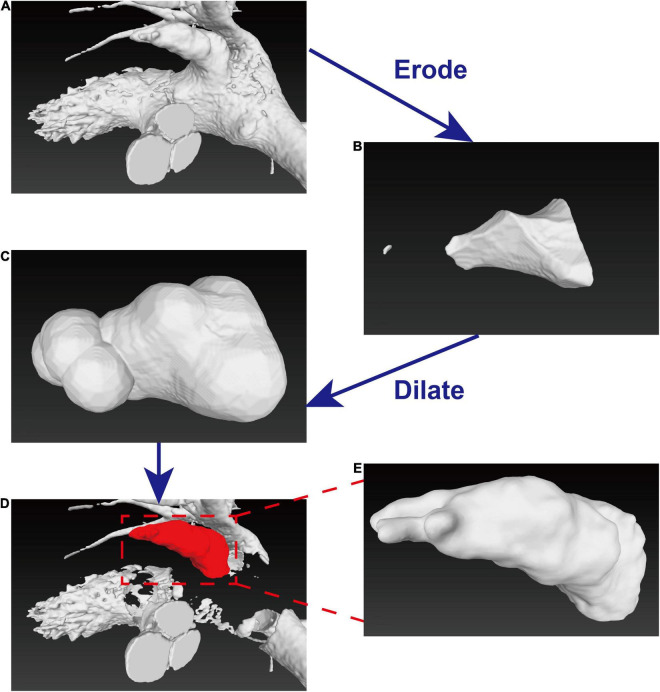
Diagram of erosion-dilation image processing. **(A–C)** Process of erode-dilation operation to obtain the approximate position of the left atrium. **(D)** Separation of the LAA and the left atrium were separated. **(E)** The LAA model used for further analysis.

To further analyze the LAA morphology, it is necessary to separate LAA from LA. To cut the LAA accurately and objectively, we introduced erosion-dilation image processing (15) (Figure 1) technology to prevent cutting LAAs from subjective interference (16). The morphology of the LAA was assessed with reconstructed three-dimensional (3D) data, and LAA orifice diameters were measured in both the long and short axes. The specific technical route was as follows: first, to determine the position of the aorta, this study used the Hough transform to detect the circle method. Next, the aorta and LV tissues and organs were separated, and tissue model 1 was obtained as shown in Figure 2A. The corrosion operation on tissue model 1 was performed, and only a part of the left atrium tissue was retained as shown in Figure 2B. The dilate operation of the tissue shown in Figure 2B was done to obtain the approximate position of the left atrium in Figure 2C. Then, the corresponding left atrium position in tissue model 1 was cleared, and LAA and left atrium were separated as shown in Figure 2D. Finally, the LAA model was retained as shown in Figure 2E.

### Fractal Analysis

Based on an adaption of the methodology described by Captur et al. (11, 17), FD (18) was calculated from the reconstructed 3D volume of LAA *via* an in-house fractal analysis program in MATLAB (MathWorks Inc., Natick, MA, United States). The roughness/curvature of the LAA surface is associated with the complexity of the LAA volume, which can be quantitatively indicated by the value of FD. For a reconstructed LAA within a volume of *M*×*M*×*M*, we partition the 3D space into boxes with side size of *r*. The minimum number of *b*oxes that can cover the entire volume of LAA is named *N*(*r*), which is a function of *r*. Therefore, the box FD (19) *D*_*B*_,one of the most commonly used FDs, is calculated as


$$D_{B}=\lim_{r\to 0}\left[logN\left(r\right)/log\left(\frac{1}{r}\right)\right]$$

### Hemodynamic Analysis

The 3D morphological structure models of LA and LAA were obtained *via* an in-house image processing algorithm. The 3D models were discretized to consecutive mesh by using the open-source library “snappyHexMesh” for the subsequent computational fluid dynamics simulations. A pressure waveform was imposed at the inlet (i.e., pulmonary veins) (17). At the outlet (i.e., mitral valve), a time-varying velocity waveform was applied to simulate AF condition. In this study, the maximum instantaneous velocity at mitral valve was measured by echocardiography at the diastole. For patients with AF, Iosifescu (18) derived blood velocity profile across the mitral valve orifice by dividing the instantaneous flow rate by the orifice area, in which the flow rate across the mitral valve was derived from the international regulation ISO5840-1:2015 (19). Therefore, using maximum instantaneous velocity and blood velocity profile, patient-specific velocity profile at mitral valve can be identified by scaling proportionally. Rigid and no-slip conditions were set at the wall boundary.

Blood flow was numerically modeled by solving the Navier-Stokes and continuity equations using the open-source library OpenFOAM version 18.04. Blood was assumed to be an incompressible and Newtonian fluid with density (ρ1,060*kg*/m^3^) and viscosity (μ0.0035Pa⋅s). The interfoam solver was used to analyze the blood flow process. The duration of each cardiac cycle was 0.8 s. Five complete cardiac cycles were taken to simulate the blood flow. Subsequently, the results in the last cardiac cycle were selected for statistical data analysis. Furthermore, to quantitatively characterize the blood washout in LAA, the blood in LA and LAA was considered as phase 1 at the end of one filling phase, whereas phase 2 was regarded as the blood flowing into the LA from the pulmonary veins at the following cardiac cycles. Then, the volume-of-fluid model was applied to estimate the volume fraction of blood residual and locate the residual blood at the end of each cardiac cycle. Finally, through the parameterization study of the morphological structure and hemodynamics index for the LAA, the essential hemodynamic characteristics, such as velocity, were obtained (20–22). We introduced residual blood flow fraction to describe the blood flow stasis. BL1/BT1 was defined as the ratio of residual blood volume to LAA/LA volume at the end of the first cardiac cycle. Additionally, more advanced hemodynamics descriptors potentially associated with the risk of thrombosis are estimated. They are time-averaged wall shear stress (TAWSS), oscillatory shear index (OSI), relative residence time (RTT), and endothelial cell activation potential (ECAP) (23–25).

### Statistical Analysis

The distribution of all continuous variables was assessed for normality using the Shapiro-Wilk test and presented as mean ± standard deviation (SD) or median (Q1, Q3), appropriately. Independent *t*-tests or Mann-Whitney *U*-tests were performed appropriately to compare the differences of scale or ordinal variables between groups. For nominal variables, the *chi*-square test and Fisher’s exact test were performed to compare them between groups. A comparison of echocardiographic, CT variables, and box-counting dimension between four types of LAA morphology was performed by one-way ANOVA followed by Tukey’s *post-hoc* test. Receiver-operating characteristic (ROC) curve analysis with the use of Youden’s index was conducted to obtain the optimum cutoff for the box-counting dimension. The comparison of the area under the curve (AUC) in ROC analysis was conducted by DeLong test (26). ANOVA was used to test whether the regression coefficient in linear regression equation is 0. Two-sided *p*-values < 0.05 were considered statistically significant. All statistical analyses were conducted with the SPSS software (version 24.0, IBM SPSS Statistics) and Stata/SE software (version 14.1, TX, United States).

## Results

### Relevant Clinical Characteristics

As shown in Table 1, a total of 46 patients were enrolled in this study as the training cohort. All 46 patients were divided into two groups based on the presence of LAA thrombi. The general baseline characteristics were compared between the two groups. Overall, the mean age was 55.8 ± 7.2 years, and 34 (73.9%) were men, with a sex ratio of 2.8:1. There were 24 patients with paroxysmal AF and 22 with persistent AF, which did not present a significant difference in the two groups. Mean body mass index (BMI) of the patients with AF and with and without thrombosis were 27.0 ± 3.7 and 25.3 ± 3.0, respectively. No statistical difference was observed in the duration of AF between the two groups of patients.

**TABLE 1:** Clinical characteristics of patients with AF and with and without thrombosis.

	Total	AF without thrombosis	AF with thrombosis	*P*-value
N	46	24	22	
Age, years	55.8 ± 7.2	54.9 ± 5.9	56.7 ± 8.4	0.398
**Sex, *n* (%)**				
Female	12 (26.1)	8 (33.3)	4 (18.7)	0.242
Male	34 (73.9)	16 (66.7)	18 (81.8)	
**Type of AF, *n* (%)**				
Paroxysmal	24 (52.2%)	13 (54.2%)	11 (50.0%)	0.777
Persistent	22 (47.8%)	11 (45.8%)	11 (50.0%)	
Height, m	1.72 (1.64, 1.76)	1.69 (1.62, 1.75)	1.75 (1.70, 1.76)	0.155
Weight, kg	76.3 ± 13.6	72.8 ± 13.2	80.1 ± 13.3	0.071
BMI, kg/m^2^	26.1 ± 3.4	25.3 ± 3.0	27.0 ± 3.7	0.105
Duration of AF, years	3.0 (2.0, 6.0)	4.0 (2.0, 6.0)	3.0 (1.8, 4.3)	0.219
**Laboratory findings**				
Triglyceride, mmol/L	1.7 ± 0.9	1.6 ± 0.7	1.8 ± 1.1	0.638
Total cholesterol, mmol/L	4.6 ± 1.2	4.6 ± 1.2	4.5 ± 1.3	0.783
**Image examination**				
LA diameter, mm	40.0 ± 5.4	38.6 ± 4.6	41.5 ± 5.9	0.069
LVEF, %	62.7 ± 6.3	63.5 ± 5.6	61.7 ± 6.9	0.332
Mean LAA orifice diameter, mm	24.0 ± 5.8	22.6 ± 6.5	25.6 ± 4.6	0.078
LAA orifice area, mm^2^	1821.7 (1122.5, 2508.9)	1331.5 (764.5, 2455.6)	2052.0 (1653.1, 2533.3)	0.082
LAA volume, cm^3^	8.2 (5.2, 11.2)	6.2 (4.6, 9.4)	10.2 (7.7, 12.0)	0.059
Hypertension, *n* (%)	18 (39.1)	6 (25.0)	12 (54.5)	0.040*
Hyperlipidemia, *n* (%)	10 (21.7)	3 (12.5)	7 (31.8)	0.159
Diabetes mellitus, *n* (%)	3 (6.5)	0 (0)	3 (13.6)	0.101
Stroke, *n* (%)	8 (17.4)	1 (4.2)	7 (31.8)	0.020*
CAD, *n* (%)	3 (6.5)	1 (4.2)	2 (9.1)	0.600
Smoke, *n* (%)	18 (39.1)	7 (29.2)	11 (50.0)	0.148
Warfarin use^†^, *n* (%)	42 (91.3)	22 (91.7)	20 (90.9)	1.000
**CHA_2_DS_2_-VaSc score, *n* (%)**				
High risk: ≥ 2	11 (23.9)	2 (8.3)	9 (40.9)	0.010*
Moderate risk: = 1	21 (45.7)	10 (41.7)	11 (50.0)	0.571
Low risk: = 0	14 (30.4)	12 (50.0)	2 (9.1)	0.003*

*Values are expressed in mean ± SD, number (percentage, %) or median (Q1, Q3).*

*Marked significant differences.

*^†^“Warfarin use” refers to the use of warfarin after obtaining the image data required for the study rather than warfarin on admission.*

*AF, atrial fibrillation; BMI, body mass index; LA, left atrium; LVEF, left ventricle ejection fraction; LAA, left atrial appendage; CAD, coronary artery disease.*

Patients with AF and thrombosis had a larger LA diameter and less left ventricle ejection fraction (LVEF) numbers, whereas neither of them was significant. However, the prevalence of hypertension was markedly lower among patients with AF and without thrombosis than that among patients with thrombosis (25.0% vs. 54.5%, *p* = 0.040). The incidence of hyperlipidemia and diabetes mellitus also manifested as such a trend, but no significant difference was observed between the two groups.

According to the CHA_2_DS_2_-VaSc score, all patients with AF were evaluated as high risk (≥ 2), moderate risk (= 1), or low risk (= 0). The rate of thrombosis in patients with AF was significantly different in both high-risk and low-risk patients. However, for moderate-risk patients, the CHA_2_DS_2_-VaSc score did not present any difference between the two groups. In addition, warfarin was used in 42 (91.3%) patients in the follow-up treatment, including 22 (91.7%) patients without LAA thrombi.

### Comparison Between Four Types of Left Atrial Appendage Morphology and Fractal Dimension

**FIGURE 3:**
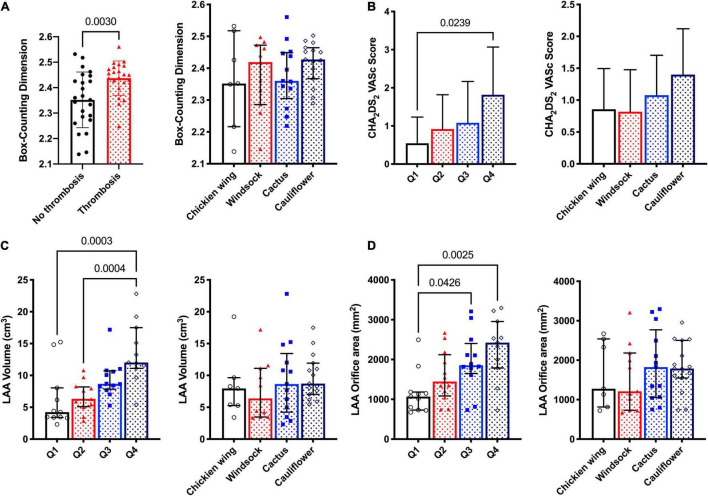
Comparison of box-counting dimension and other LAA parameters. **(A)** The LAA box-counting dimension of patients with AF and thrombi was significantly higher than that of patients without thrombi (*p* = 0.003), yet the box-counting dimension was not statistically different between the four types of LAA. **(B)** Comparison of CHA_2_DS_2_-VaSc between four groups divided by quartile indicated that the CHA_2_DS_2_-VaSc score was markedly higher in Q4 group compared to Q1 group, while it appeared no significant difference between four types of LAA. **(C)** LAA volumes of patients in Q4 were dramatically larger than those of patients in the Q1 and Q2 groups, and no difference was observed in four types of LAA. **(D)** Similar to LAA volume, the LAA orifice area was also significantly larger in the Q4 group.

The box-counting dimensions of LAAs were compared to test the hypothesis that patients with AF and thrombosis would have a higher FD. The results suggested that the LAA FD of patients with AF and thrombi was significantly higher than those of patients without thrombi (2.42 ± 0.072 vs. 2.35 ± 0.109, *p* = 0.003) (Figure 3A). To further investigate the potential differences between four types of LAA morphology, we compared the crucial imaging parameters and box-counting dimensions (Figure 3 and Supplementary Table 1). None of the echocardiographic and CT variables were significantly different among the four groups, nor was thrombosis and the box-counting dimension (Figure 3A), indicating that this classification might not reflect LAA complexity. In addition, we compared LAA volume, orifice area, and CHA_2_DS_2_-VaSc score between the four morphological classifications and between the groups divided by FD quartiles. Interestingly, the volume and orifice area of LAA markedly increased in the group with higher FD; however, they did not differ significantly among the four LAA morphological classifications (Figures 3C,D). In addition, the CHA_2_DS_2_-VaSc score was dramatically higher in Q4 than that in Q1, while there seemed to be no difference among the four LAA morphological classifications (Figure 3B).

### Fractal Dimension Demonstrated the Relationship Between Morphological Complexity and Stroke

To further illustrate the profound relationship between LAA morphological complexity and the long-term prognosis of patients with AF, an independent validation cohort were enrolled and were divided into two groups based on whether they had stroke after the diagnosis of AF. There were 338 patients without stroke and 105 patients with stroke (Table 2). The median age of patients with AF and stroke was markedly lower than that of patients without stoke [55.0 (45.0, 64.0) vs. 58.0 (51.0, 63.0), *p* = 0.045]. In addition, there were no statistical differences in gender composition and the incidences of hypertension, diabetes, and vascular disease between the two groups. CHA_2_DS_2_-VaSc score was significantly different between the two groups. Interestingly, the average box-counting dimension of patients with stroke was dramatically higher than that of patients without stroke (2.45 ± 0.08 vs. 2.41 ± 0.09, *p* = 0.001), further suggesting that the degree of morphological complexity of LAA might be closely related to cerebral infarction complicated by AF.

**TABLE 2:** Clinical characteristics of patients with AF and with and without stroke.

	Total	AF without stroke	AF with stroke	*P*-value
N	443	338	105	
Age, years	58.0 (50.0, 63.0)	58.0 (51.0, 63.0)	55.0 (45.0, 64.0)	0.045*
**Sex, *n* (%)**				
Female	135 (30.5)	110 (32.5)	25 (23.8)	0.089
Male	308 (69.5)	228 (67.5)	80 (76.2)	
Hypertension, *n* (%)	212 (47.9)	159 (47.0)	53 (50.5)	0.538
Congestive heart failure, *n* (%)	23 (5.2)	16 (4.7)	7 (6.7)	0.436
Diabetes mellitus, *n* (%)	75 (16.9)	63 (18.6)	12 (11.4)	0.085
Vascular disease, *n* (%)	39 (8.8)	26 (7.7)	13 (12.4)	0.139
**CHA_2_DS_2_-VaSc score, *n* (%)**				
High risk: ≥ 2	245 (55.3)	147 (43.5)	98 (93.3)	<0.001*
Moderate risk: = 1	114 (25.7)	109 (32.2)	5 (4.8)	<0.001*
Low risk: = 0	84 (19.0)	82 (24.3)	2 (1.9)	<0.001*
Box-counting dimension	2.43 ± 0.09	2.41 ± 0.09	2.45 ± 0.08	0.001*

*Values are expressed in mean ± SD, number (percentage, %) or median (Q1, Q3).*

*Marked significant differences.

*AF, atrial fibrillation.*

### Diagnostic Accuracy of Box-Counting Dimension in Training and Validation Cohorts

**FIGURE 4:**
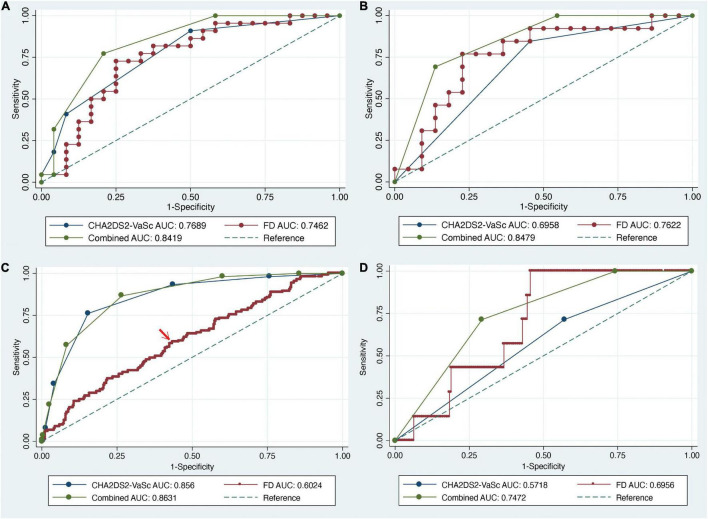
ROC analysis of FD and CHA_2_DS_2_-VaSc score in training and validation cohorts. **(A)** ROC curve for assessing the patients in the training cohorts. **(B)** ROC analysis of low- to moderate-risk patients with AF. Both AUC of FD and combined scoring system were significantly larger than that of CHA_2_DS_2_-VaSc score (*p* = 0.005 and 0.001, respectively). **(C)** ROC analysis of FD and CHA_2_DS_2_-VaSc score among all patients in the validation cohort. **(D)** ROC analysis in low- to moderate-risk patients in the validation cohort.

To further investigate the relationship between thrombosis and LAA morphology complexity, ROC analysis was performed to reveal the accuracy of box-counting dimensions in diagnosis of LAA thrombus and stroke in the training and validation cohort, respectively (Table 3 and Figure 4). The optimum diagnostic thresholds for thrombosis and stroke in patients with AF were 2.426 and 2.438, respectively (Figures 4A,C). Through ROC analysis, the AUC of the box-counting dimension was 0.7462. The AUC of FD was slightly lower than that of the CHA_2_DS_2_-VaSc score (0.7462 vs. 0.7689, *p* = 0.811). However, for low- to moderate-risk patients, the diagnostic accuracy of FD was higher than the CHA_2_DS_2_-VaSc score (AUC 0.7622 vs. 0.6958, *p* = 0.545) (Figure 4B). We consequently combined the shape complexity of LAA and CHA_2_DS_2_-VaSc and made it into a new risk scoring system. To be specific, box-counting dimensions greater than 2.426 were awarded 1 point in the training cohorts, otherwise 0 point. The combined risk score was significantly more accurate in the diagnosis of LAA thrombus than the CHA_2_DS_2_-VaSc scoring system alone in low- to moderate-risk patients (AUC 0.8479 vs. 0.6958, *p* = 0.009). The same trend was also observed among all patients, whereas it was not statistically significant. Similarly, box-counting dimensions more than 2.438 were given one more point to be added to the CHA_2_DS_2_-VaSc score. In addition, the ROC analysis indicated that the diagnostic accuracy of combined risk score was higher than that of CHA_2_DS_2_-VaSc score alone among all patients and low- to moderate-risk patients (Figures 4C,D).

**TABLE 3:** ROC analysis of patients with AF in the training and validation cohorts.

	Training cohort (*n* = 46)			Validation cohort (*n* = 443)		
		*P*-value			*P*-value^#^	
	AUC (95% CI)	Compared to 0.5	Compared to AUC of CHA_2_DS_2_-VaSc	AUC (95% CI)	Compared to 0.5	Compared to AUC of CHA_2_DS_2_-VaSc
All patients						
FD	0.746 (0.599, 0.893)	0.004*	0.811	0.602 (0.541, 0.663)	0.002*	<0.001*
CHA_2_DS_2_-VaSc	0.769 (0.643, 0.895)	0.002*	-	0.856 (0.817, 0.895)	<0.001*	-
Combined^†^	0.842 (0.732, 0.952)	<0.001*	0.063	0.863 (0.827, 0.899)	<0.001	0.427
Low to moderate risk						
Number of patients n (%)	35 (76.1)			198 (44.7)		
FD	0.762 (0.592, 0.932)	0.005*	0.545	0.696 (0.568, 0.823)	0.079	0.171
CHA_2_DS_2_-VaSc	0.696 (0.548, 0.843)	0.056	-	0.572 (0.388, 0.756)	0.519	-
Combined^†^	0.848 (0.733, 0.963	0.001*	0.009*	0.747 (0.608, 0.886)	0.026*	<0.001*

*p-Values were obtained by z-test.*

*Marked significant differences.

*^†^If the box-counting dimension value is greater than threshold (2.426 in the training cohort; 2.438 in the validation cohort), one point will be added to CHA_2_DS_2_-VaSc system, and the new risk score was assessed by ROC analysis.*

*^#^The DeLong test was used to compare the AUCs.*

*AUC, area under the curve; CI, confidential interval; FD, fractal dimension (represented by box-counting dimension).*

### Relationship Between Fractal Dimension and Left Atrial /Left Atrial Appendage Parameters

**FIGURE 5:**
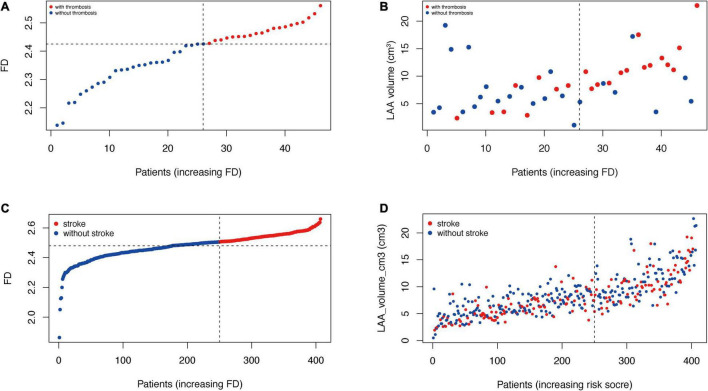
Risk plot of training and validation cohort. **(A,C)** LAA box-counting dimension of each patient is plotted and presents the cutoff value that defines high- and low-risk patients in the training and validation cohorts, respectively. **(B,D)** Each patient’s LAA volume is plotted with its box-counting dimension, suggesting the positive correlation between LAA volume and morphological complexity.

**FIGURE 6:**
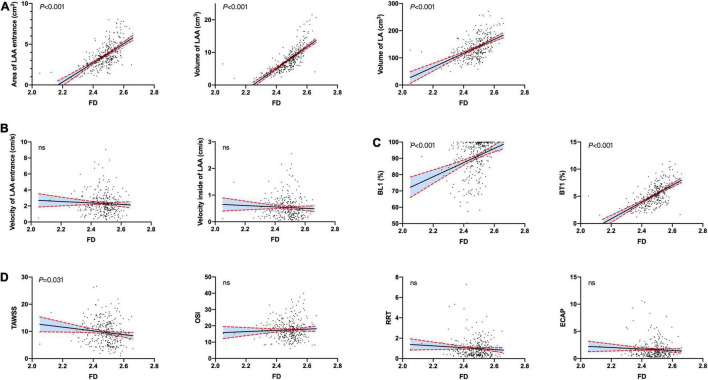
Linear regression of FD and hemodynamic parameters. **(A)** The correlation between FD and LAA/LA morphological parameter. **(B)** The correlation between FD and blood flow velocity in LAA. **(C)** FD was positively related to the ratio of residual blood volume to LAA/LA volume at the end of the first cardiac cycle. **(D)** FD was markedly negatively related to TAWSS. BL1%: Ratio of the volume of blood remaining in the LAA to the volume of the LAA at the end of first cardiac cycle. BT1%: Ratio of the volume of blood remaining in the LAA to the volume of the entire left atrium at the end of the first cardiac cycle.

We found that the morphological complexity of LAA was positively correlated with LAAs’ orifice area and volume (Figures 3C,D). We plotted a scatter diagram to reflect this trend (Figures 5A,C). It shows that, with an increase in the volume of LAA, the degree of morphological complexity gradually increases in both training and validation cohorts, so does the risk of thrombosis and stroke (Figures 5B,D). Furthermore, we performed simple linear regression for FD and LA/LAA parameters (Figures 6A,B). FD showed significant positive correlation with the area of LAA entrance, LAA, and LA volume (Figure 6A), yet the correlation of FD and the blood flow velocity inside LAA was not identified (Figure 6B).

### Hemodynamic Characteristics of Left Atrial Appendage and Thrombosis

To further evaluate the hemodynamic characteristics in LAAs of patients with AF and with and without thrombi, we obtained a hemodynamic model of the LAA through parameterization studies. It suggested that LAAs with thrombi and higher FD were prone to blood stasis and lower blood flow rate (Supplementary Figure 2 and Figure 5). Furthermore, the residual blood volume in LAAs of patients without thrombosis was lower than those of patients with thrombosis after the sixth cardiac cycle, and the distribution of residual blood volume was more dispersed in LAAs without thrombi (Supplementary Figure 3). Linear regression between FD and BL1% or BT1% indicates the markedly positive correlation (Figure 6C). In addition, TAWSS was negatively correlated with FD, whereas the linear correlations between FD and OSI, RRT, and ECAP were not significant (Figure 6D).

## Discussion

Our study takes the first step in assessing the thrombosis and stroke among patients with AF from the perspective of LAA morphological complexity, which was quantitatively analyzed by means of fractal geometry. It is technically feasible to calculate FD of LAAs through enhanced CT images of patients with AF. Our data suggested that patients with AF having higher box-counting dimension were more prone to LAA thrombi and stroke. Furthermore, FD of LAA showed more exceptional ability to diagnose thrombosis and stroke than the CHA_2_DS_2_-VaSc scoring system among low- to moderate-risk patients with AF. In addition, hemodynamic analysis of LAAs indicated that blood flow rate was lower in LAAs with thrombi and higher with box-counting dimension. This study revealed the relationship between the thrombosis and morphological complexity of LAA in patients with AF and provided additional clinical implications for risk stratification.

The relationship between the shape of LAAs and thrombosis has been explored for a long time, and previous studies have reached a consensus that LAA morphology is correlated with the risk of thrombus formation and stroke or transient ischemia attack in patients with AF (27–29). It has been reported that LAA volume, LAA empty velocity, and LAA orifice area could be used to predict thrombus formation (27, 29), yet these parameters did not present significant differences between the two groups in our cohort. Interestingly, according to the study by Yamamoto et al. (27), most of the patients with LAA thrombus had ≥ 3 LAA lobes, whereas the thrombi were observed in only 0.7% of patients with AF and with one or two LAA lobes. These studies indicated that thrombosis was closely related to the complexity of the LAA. Correspondingly, our data quantitatively evaluated the morphological complexity of LAA and further confirmed the correlation between the LAA thrombosis and morphological complexity. In contrast, Fukushima et al. demonstrated the relationship between LAA flow velocity (LAAFV) and LAA morphology in patients with paroxysmal AF based on the four-type classification system of LAA morphology, indicating that LAAFV was significantly higher in patients with chicken wing LAA (5). Likewise, another study pointed out that patients with chicken wing morphology were less prone to cerebral embolic events (28), which indicated that the higher LAAFV might be associated with less thrombus formation and stroke. These data suggest that LAA morphology can significantly affect the state of blood flow within LAA, and thus further lead to thromboembolism (30, 31). The traditional four-type morphological classification of LAA can reflect the association between LAA morphology and thrombosis or embolic event. However, this classification system cannot quantitatively assess the complexity of LAA, thus there were contrary conclusions about the relationship between LAA morphology and thrombosis or stroke in the previous studies. Consequently, we provided a novel method to evaluate the morphological complexity of both outer and internal sides of LAA (5, 28, 32, 33).

The fractal geometry analytic method proposed in this study can accurately quantify the morphological complexity of LAAs. A recent study revealed that complex LAA morphology characterized by an increased number of LAA lobes was associated with the presence of LAA thrombi and was an independent predictive factor in thrombosis (27). Blood stasis caused by more lobes of LAA may be an essential factor in the formation of thrombosis. Accordingly, the morphological complexity inside the LAA, which directly determines the hemodynamic state of blood in the LAA, might reflect the relationship between LAA morphology and blood flow more intuitively. Moreover, the hemodynamic model of LAAs reached a similar conclusion: the blood flow in the LAA with a high FD and thrombosis was slower, prone to stasis and higher RRT, and residual blood volume fraction in the LAA after six cardiac cycles was higher than that of the control group.

Through ROC analysis, we identified the cutoff values of box-counting dimension as 2.426 and 2.438 in training and validation cohorts, indicating that patients with FD values higher than thresholds should be considered for active treatment, including anticoagulant therapy. The CHA_2_DS_2_-VaSc score system, widely used to evaluate the thrombosis risk among patients with AF, was reported to have limited efficacy in predicting thrombosis and stroke in patients with AF and low to moderate risk of thrombosis (34, 35). Therefore, it remains controversial whether active treatment should be performed among these patients. Our findings imply that FD had more potency to evaluate the possibility of both thrombosis and stroke in low- to moderate-risk patients with AF, which might be a clinical parameter that can be used to assess these patients. Besides, FD indirectly compensates for blood flow stasis that is not covered by the CHA_2_DS_2_-VaSc score, and the novel scoring system, combining the FD and CHA_2_DS_2_-VaSc score, presents higher accuracy in diagnosis of LAA thrombosis.

At present, patients with AF are routinely treated with antiplatelet or anticoagulant therapy, and 41 (91.3%) patients in our study took warfarin. To altogether remove the site of thrombus, some other patients underwent LAA. Although a vital part of LA, LAA is involved in the contraction and endocrine function of LA. Considering the side effects of warfarin and the decreased LA compliance after LAA (33), anticoagulation or surgery needs to be implemented more cautiously in patients with AF. The FD of LAA can be added to the CHA_2_DS_2_-VaSc scoring system to more accurately stratify patients with stroke and guide treatment decisions. Therefore, further research is needed to confirm clinically that the LAA FD is related to the occurrence of stroke.

There are several limitations to our study. First, the relatively small sample size collected from our single center and the retrospective nature might cause some bias in the comparison results. The starting point of the disease course of patients with AF is mainly through the patient’s recollection and health examination report, which may have an impact on the course of the patient’s illness. Secondly, the diagnosis of LAA thrombus in the training cohort largely depends on TEE images rather than pathological or surgical examination. Poor quality ultrasound images of patients cannot determine whether the patients had LAA thrombi, thereby leading to a certain amount of error. Due to the limitations of retrospective data acquisition, the division of patients in the two cohorts is not random, which may lead to a certain selection bias. In addition, the onset and duration of patients with persistent atrial fibrillation (PAF) could not be accurately recalled and recorded by these patients, which may also affect the results to some extent. Moreover, an absence of anticoagulants in the treatment of included patients before CT or TEE could be a confounding factor in the thrombosis and stroke. In addition, because the contrast agent may not be completely adherent during scanning, the 3D reconstructed model cannot wholly reflect the internal shape of the patient’s LAA. Therefore, a prospective study, including large samples, should be conducted to reduce the biases of the result and to verify the conclusion of this study further.

## Conclusion

The LAA morphological complexity is closely associated with thrombosis and stroke in patients with AF. It can be quantitatively evaluated with box-counting dimension calculated by enhancing the CT image and our fractal analysis approaches. Moreover, FD presented higher accuracy than the CHA_2_DS_2_-VaSc score in the diagnosis of LAA thrombosis and stroke in low- to moderate-risk patients with AF. Furthermore, preliminary hemodynamic studies have confirmed that complicated LAA morphology is more likely to cause blood flow stasis and thrombosis. The threshold of the box-counting dimension was defined, and a new combined scoring system was established for better risk assessment, potentially providing an efficient approach that may better assess the necessity for anticoagulation in low- to moderate-risk patients with AF.

## Data Availability Statement

The raw data supporting the conclusions of this article will be made available by the authors, without undue reservation.

## Ethics Statement

The studies involving human participants were reviewed and approved by Institutional Review Board at the FuWai Hospital. Written informed consent for participation was not required for this study in accordance with the national legislation and the institutional requirements. Written informed consent was obtained from the individual(s) for the publication of any potentially identifiable images or data included in this article.

## Author Contributions

ZZ, HF, and YY participated in the design of the study. CL, QG, RW, and QL conducted the statistical analysis and drafted the manuscript. XL and LW participated in hemodynamic analysis and helped to draft the manuscript. RW carried out important literature review and modified the manuscript. All authors read and approved the final manuscript.

## Conflict of Interest

RW, QL, and XL were employed by the Hangzhou Shengshi Technology Co., Ltd. The remaining authors declare that the research was conducted in the absence of any commercial or financial relationships that could be construed as a potential conflict of interest.

## Publisher’s Note

All claims expressed in this article are solely those of the authors and do not necessarily represent those of their affiliated organizations, or those of the publisher, the editors and the reviewers. Any product that may be evaluated in this article, or claim that may be made by its manufacturer, is not guaranteed or endorsed by the publisher.
