# Supplementary material for: Fractal Geometry Illustrated Left Atrial Appendage Morphology That Predicted Thrombosis and Stroke in Patients With Atrial Fibrillation
Source: Front Cardiovasc Med. 2022 May 10;9:779528. doi: 10.3389/fcvm.2022.779528 (PMC9127617; doi:10.3389/fcvm.2022.779528)
Supplement: Supplementary file 1 [file Data_Sheet_1.docx]

**Supplemental Materials**

**Title:** Fractal geometry illustrated left atrial appendage morphology which predicted thrombosis and stroke in atrial fibrillation patients

**Authors:** Chuxiang Lei^1^, Qi Gao^2^, Runjie Wei^2^, Qijie Li^3^, Xingli Liu^3^, Lingmin Wu^1^, Yan Yao^1^, Hongguang Fan^1, †^, Zhe Zheng^1, †^.

**Affiliations**

1. Department of Cardiac Surgery, State Key Laboratory of Cardiovascular Diseases, National Center for Cardiovascular Diseases, Fuwai Hospital, Peking Union Medical College and Chinese Academy of Medical Sciences, Beijing, 100037, China.

2. Institute of Fluid Engineering, School of Aeronautics and Astronautics, Zhejiang University, Hangzhou, Zhejiang province, 310027, China

3. Hangzhou Shengshi Tech. Inc., Hangzhou, Zhejiang province, 311199, China

† Corresponding authors for this work

**Co-Correspondence**

Hongguang Fan

Department of Cardiac Surgery, State Key Laboratory of Cardiovascular Diseases, National Center for Cardiovascular Diseases, Fuwai Hospital, Peking Union Medical College and Chinese Academy of Medical Sciences. No. 167, North Lishi, Road, Xicheng District, Beijing, 100037, China.

TEL: +86 10 8839 6665

Fax number: 00861088396665

Email: [fanhongguang@fuwai.com](mailto:fanhongguang@fuwai.com)

Zhe Zheng

Department of Cardiac Surgery, State Key Laboratory of Cardiovascular Diseases, National Center for Cardiovascular Diseases, Fuwai Hospital, Peking Union Medical College and Chinese Academy of Medical Sciences. No. 167, North Lishi, Road, Xicheng District, Beijing, 100037, China.

TEL: +86 10 8839 8265

Fax number: 00861088398265

Email: [zhengzhe@fuwai.com](mailto:zhengzhe@fuwai.com)

*Supplemental Table S1*. Comparison of echocardiographic and computed tomography variables between 4 types of LAA morphology. Values are expressed in mean ± SD and number (percentage, %).

|  | Chicken wing | windsock | cactus | cauliflower | P value |
| --- | --- | --- | --- | --- | --- |
| Thrombosis, n (%) | 7 (15.2) | 11 (23.9) | 13 (28.3) | 15 (32.6) | 0.248^†^ |
| LVEF (%) | 65.4 ± 7.7 | 61.6 ± 7.8 | 60.2 ±5.4 | 64.4 ± 4.2 | 0.175 |
| LAA orifice area, mm^2^ | 1926.6 ± 971.7 | 1558.4 ± 828.7 | 2098.2 ± 1060.7 | 1828.2 ± 640.8 | 0.509 |
| LAA volume, cm^3^ | 8.5 ± 5.2 | 7.6 ± 4.5 | 9.4 ± 5.8 | 9.2 ± 4.2 | 0.798 |
| Box-counting dimension | 2.36 ± 0.15 | 2.38 ± 0.11 | 2.38 ± 0.10 | 2.42 ± 0.06 | 0.451 |

Values are mean ± standard deviation, or number (%). *P*-values for continuous variables were obtained by one-way ANOVA test.

† P-values for nominal variables were obtained by Chi-square test.

LVEF: left ventricle ejection fraction; LAA: left atrial appendage.


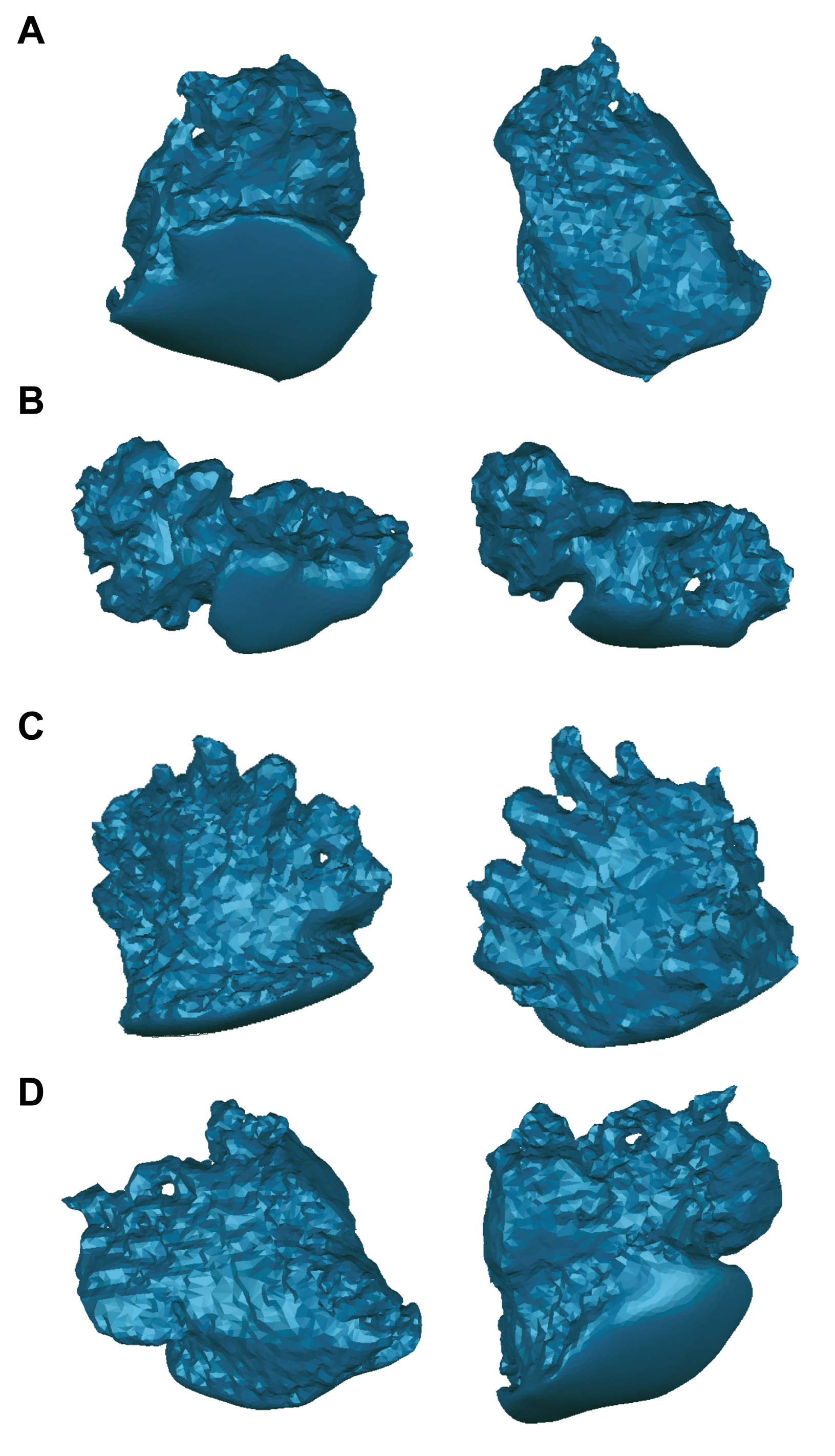


**Supplemental Figure S1. Classification of LAA morphology.** Representative three-dimensional reconstruction images of each type of LAA are shown. A. Chicken wing; B. Windsock; C. Cactus; D. Cauliflower.


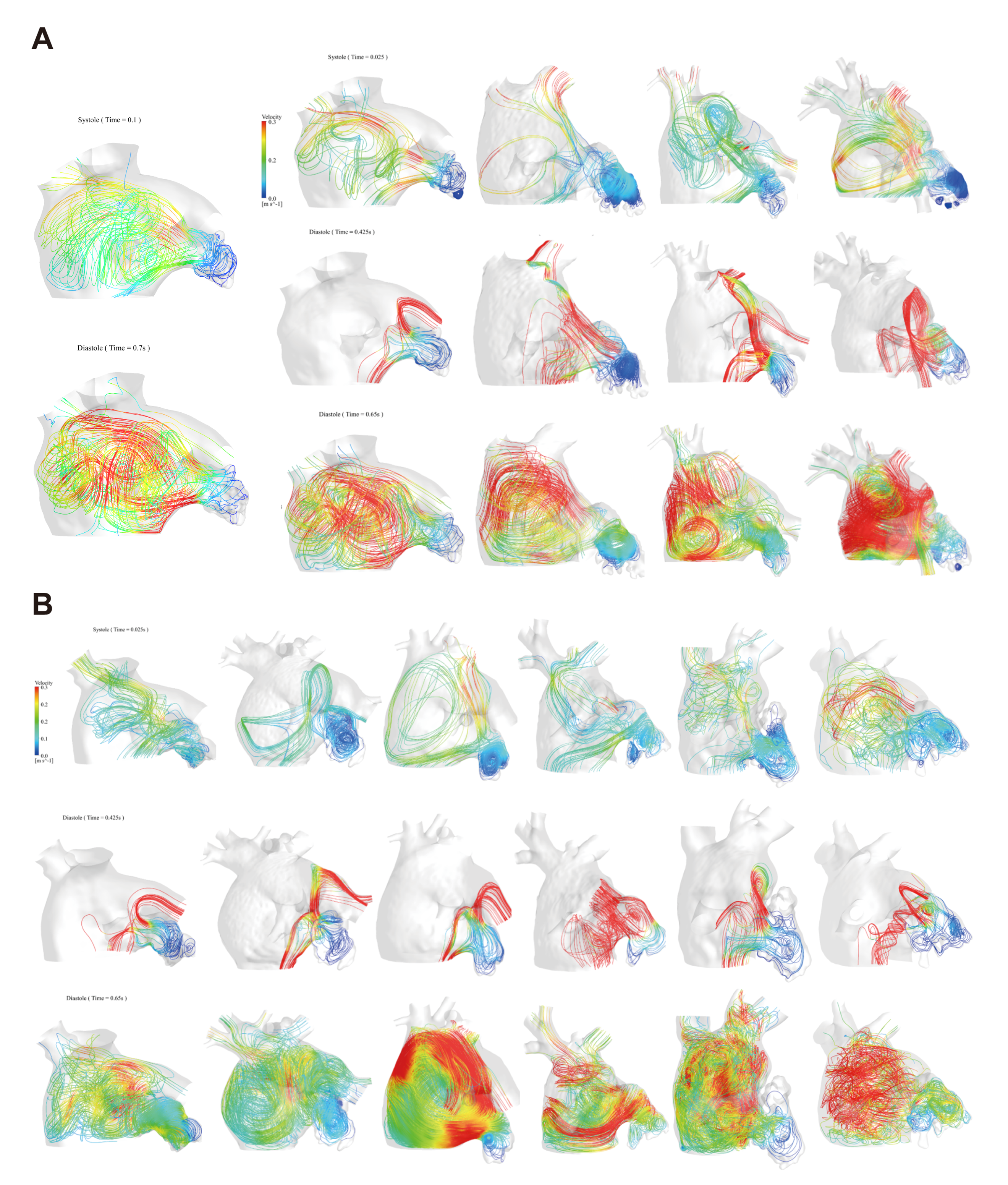


**Supplemental Figure S2.** **Computer simulation of the blood flow line in LAA with (A) and without (B) thrombi.**

**
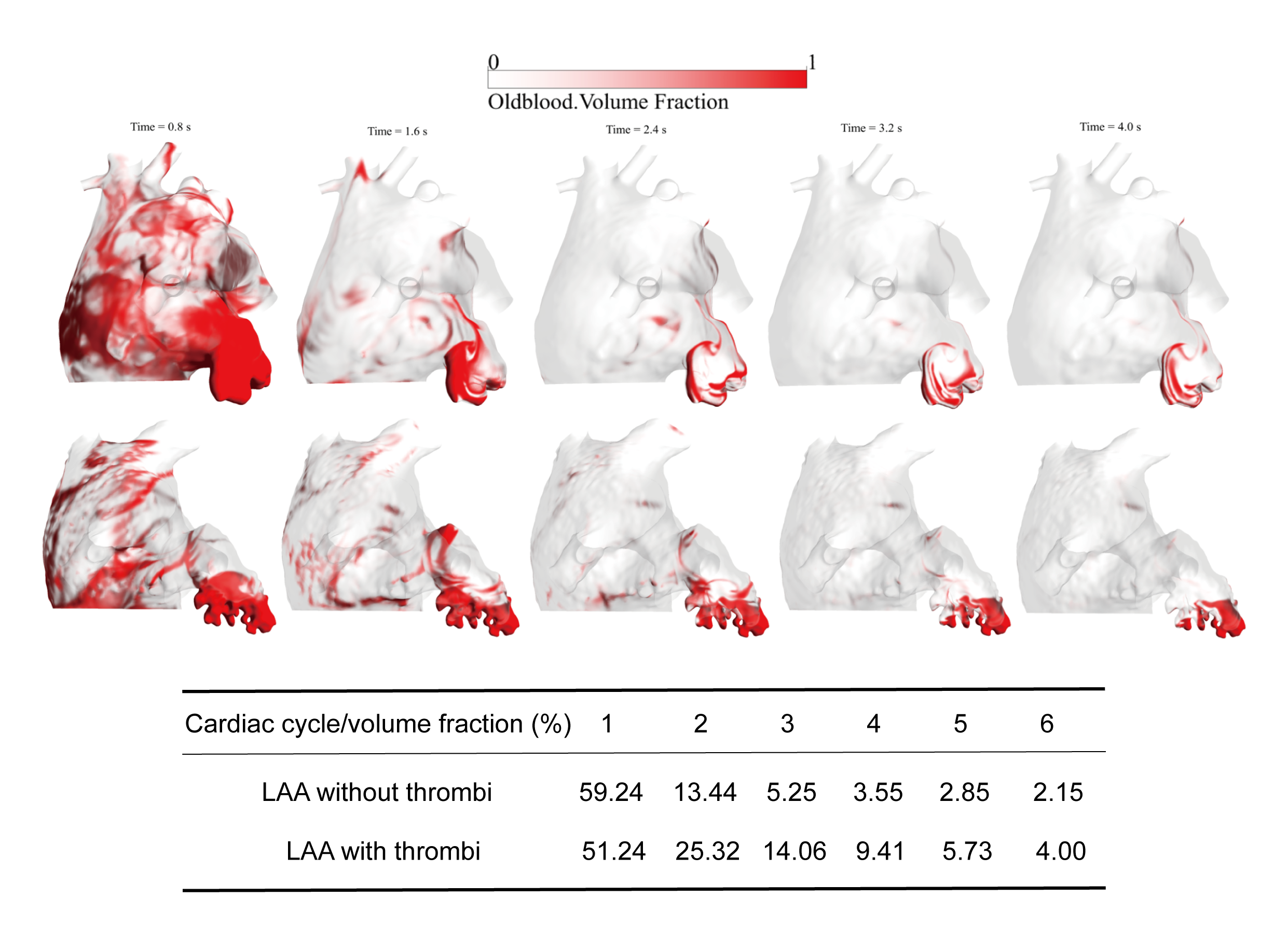
**

**Supplemental Figure S3.** **Computer-simulated residual blood volume fraction of the LAAs in 6 consecutive cardiac cycles.** The residual blood volume of LAA with thrombi (A) was higher than that of LAA without thrombi (B).

**Supplemental Methods**

*Classification of LAA morphology*

According to the previous study reported by Kimura et al.(1), the classification of LAA morphology discriminated by the computed tomography measurements of the length, angle, and number of lobes of the LAA, and LAAs were classified into four types including chicken wing, windsock, cactus, and cauliflower. In this classification system, LAAs were classiﬁed into the following four types: (1) ‘chicken wing’: a main lobe of more than 4cm long, with a folded angle of less than 100 degrees (Supplemental Figure S1A); (2) ‘windsock’: a main lobe of more than 4cm long, with a folded angle of more than 100 degrees (Supplemental Figure S1B); (3) ‘cactus’: a main lobe of less than 4cm long, with more than 2 lobes over 1cm (Supplemental Figure S1C); and (4) ‘cauliﬂower’: a main lobe of less than 4cm long, without any forked lobes (Supplemental Figure S1D). Two independent reviewers reconfirmed the classification of each patient.

*Cardiac CT scan and Image analysis*

Contrasted-enhanced cardiac computed tomography (CT) imaging was performed with a 64-slice CT scanner (Siemens, Biography 64) using valid technical parameters including a collimation of 64*0.6 mm, gantry rotation of 330 ms, a pitch of 0.2, a tube voltage of 120 kV, and a tube current of 770 mA. For contrast enhancement, a nonionic contrast agent was injected intravenously at a rate of 3.5 mL/second with a power injector through an 18- to 20-gauge catheter in the antecubital fossa. The time between contrast injection and maximum enhancement in the left atrium was used as the scanning delay for optimal contrast in the LAA. The 3-dimensional (3D) structures of the LA were constructed in the left atrial filling phase and late ventricular systole.

*Echocardiographic data analysis*

All the participants had received TEE within three days before the scheduled ablation procedure, which was performed with an EPIQ 7 system (Philips Healthcare, Bothell, WA, USA). The images were stored digitally for playback and analysis. Two independent reviewers reconfirmed the analysis of echocardiographic images, and all the comprehensive echocardiographic measurements by TEE conformed to the recommendations of the American Society of Echocardiography(2).

1. T. Kimura, S. Takatsuki, K. Inagawa, Y. Katsumata, T. Nishiyama, N. Nishiyama, K. Fukumoto, Y. Aizawa, Y. Tanimoto, K. Tanimoto, M. Jinzaki and K. Fukuda: Anatomical characteristics of the left atrial appendage in cardiogenic stroke with low CHADS2 scores. *Heart Rhythm*, 10(6), 921-5 (2013) doi:10.1016/j.hrthm.2013.01.036

2. R. T. Hahn, T. Abraham, M. S. Adams, C. J. Bruce, K. E. Glas, R. M. Lang, S. T. Reeves, J. S. Shanewise, S. C. Siu, W. Stewart and M. H. Picard: Guidelines for performing a comprehensive transesophageal echocardiographic examination: recommendations from the American Society of Echocardiography and the Society of Cardiovascular Anesthesiologists. *J Am Soc Echocardiogr*, 26(9), 921-64 (2013) doi:10.1016/j.echo.2013.07.009
